# Pathogenic Variants in *GALC* Gene Correlate With Late Onset Krabbe Disease and Vision Loss: Case Series and Review of Literature

**DOI:** 10.3389/fneur.2020.563724

**Published:** 2020-10-15

**Authors:** Nicholas A. Bascou, Maria L. Beltran-Quintero, Maria L. Escolar

**Affiliations:** Program for the Study of Neurodevelopment in Rare Disorders and Department of Pediatrics, Children's Hospital of Pittsburgh, University of Pittsburgh Medical Center, Pittsburgh, PA, United States

**Keywords:** neurodevelopment, Krabbe disease (globoid cell leukodystrophy), vision loss, genotype-phenotype correlation, later-onset

## Abstract

**Background:** Krabbe disease is an autosomal recessive demyelinating disorder resulting from deficiency of the lysosomal enzyme galactocerebrosidase. While blindness is often described as a characteristic finding of the disease, it is more common in the infantile phenotype, where vision loss typically arises in the late stages of disease. In comparison, reports of vision loss in late onset phenotypes are less well-described and may be subject to variation between genotypes.

**Methods:** Charts of Krabbe patients with a confirmed diagnosis, who presented with substantial visual impairment, were retrospectively reviewed from a larger group of 199 Krabbe patients. Assessment of clinical status was obtained through review of neurological evaluations, neurodevelopmental assessments, ophthalmological evaluations, visual evoked potentials (VEP), electroretinogram (ERG), nerve conduction velocity (NCV) studies, auditory brainstem responses (ABR), and brain magnetic resonance imaging.

**Results:** Five late onset patients with Krabbe disease (four juvenile and one late-infantile) were included. Three patients were homozygous for c.956A>G_p.Y319C, one was compound heterozygous for c.296+1G>T and c.956A>G_p.Y319C, and one was compound heterozygous for c.1186C>T_p.R396W and c.1901T>C_p.L634S. All patients were of Asian descent and presented initially with vision impairment. Notably, the patients did not present with marked appendicular spasticity or axial hypotonia and all five reached developmental milestones within the normal time frame. For neurophysiological testing, no patient showed abnormalities in NCV or ABR. However, abnormalities in VEP or ERG were seen in all patients. The one patient who underwent transplantation stabilized following treatment.

**Conclusions:** Depending on their genotype, patients with late onset Krabbe disease may initially present with vision loss. Furthermore, patients with p.L634S and p.Y319C should be closely monitored for changes in vision and VEP. This knowledge will become increasingly important as physicians may otherwise overlook these signs and symptoms when monitoring children identified through newborn screening who have the variants described in this report.

## Background

Krabbe disease, otherwise known as globoid cell leukodystrophy, is an autosomal recessive demyelinating disorder resulting from deficiency of the lysosomal enzyme galactocerebrosidase (GALC) (1–3). GALC deficiency leads to an accumulation of galactocerebroside and its parent cytotoxic compound, galactosylsphingosine, which is considered toxic to both the central and peripheral nervous system (2). Excess levels of galactocerebroside leads to the formation of multinucleated macrophages. Accumulation of galactosylsphingosine elicits two critical factors in progressive demyelination: the death of oligodendrocytes and myelin arrest (3).

The infantile phenotype (estimated as ~85% of cases) is the most common and aggressive form of the disease, with an incidence rate of about one in 100,000 (4, 5). The onset of clinical symptoms, characterized by hyperirritability, blindness, hypertonicity, intractable seizure, and intellectual delay (2, 6), occurs within the first 12 months of life (7–9). In comparison to the infantile phenotype, later onset forms are significantly less prevalent (estimated as ~15% of cases) and patients exhibit much more heterogeneity in their presentation. For example, juvenile onset, occurring between 3 and 16 years of life, is typified by cognitive regression, abnormal behaviors, and ataxia.

While blindness is often thought to be a prominent feature of infantile Krabbe disease, due to anecdotal reports of vision loss, its prevalence may be overestimated, as a recent study describing a large cohort of children found blindness in only 9% of patients (7). Although there are reports of blindness in juvenile onset patients as well, the limited number of patients with the later onset phenotype has made conducting large cohort studies difficult and is limited to case reports. Therefore, it is possible that blindness is not a ubiquitous feature of Krabbe disease but is characteristic of certain pathogenic variants and is, thus, subject to phenotype-genotype correlations.

We report here on five individuals with late onset Krabbe disease who presented with complete blindness or severely impaired vision as one of the earliest signs of disease. Four of the patients were either homozygous or heterozygous for the c.956A>G_p.Y319C variant, which has been previously reported to correlate with later onset phenotypes. The fifth patient tested positive for two separate known variants, one of which is reported to correlate with late onset (c.1901T>C_p.L634S) and the other of which disease onset is typically early (c.1186C>T_p.R396W). In addition to reporting these five novel cases, we conducted a brief literature review of previously published cases describing individuals with Krabbe disease who also tested positive for the relevant pathogenic variants.

## Methods

The study population consisted of five patients with a confirmed diagnosis of Krabbe disease who presented with late onset disease and substantial visual impairment. These patients were derived from a larger cohort of patients seen and evaluated at the Program for the Study of Neurodevelopment in Rare Disorders (NDRD) at UPMC's Children's Hospital over the past 20 years. In total, the NDRD has evaluated 199 patients for Krabbe disease, including 189 with a confirmed diagnosis and an additional 10 who screened positive on newborn screening (NBS) but have not yet developed signs of disease. These patients have traveled from all over the world; however, the majority are non-Hispanic White in respect to ethnicity; 24 were of Asian descent. Of this larger cohort, the five patients described in this study are the only ones to present with both late onset disease and the predominant symptom of vision loss. They are also the only patients evaluated at the NDRD who tested positive for either the p.Y319C or p.L634S variants. Data on a large portion of the other Krabbe patients evaluated at the NDRD can be found in Bascou et al. (7) and Beltran-Quintero et al. (8).

All diagnoses of Krabbe disease were confirmed through enzyme testing and genetic analysis. GALC levels were measured by analyzing leukocyte lysates. Although there is some disagreement between experts, values <0.50 nmol/h/mg protein are typically considered abnormal. Psychosine measurements were analyzed from fresh dried blood spots in Dr. Michael Gelb's laboratory at the University of Washington. However, at the time of measurement, normal ranges for psychosine had not yet been established. All variants were classified as “pathogenic” according to classification on diagnostic reports, with the exception of c.296+1G>T, which was classified as “potentially pathogenic.” One patient in the study received hematopoietic stem cell transplantation (HSCT). Assessment of clinical status was obtained through retrospective review of neurological evaluations, neurodevelopmental assessments, ophthalmological evaluations, visual evoked potentials (VEP), nerve conduction velocity (NCV) studies, auditory brainstem responses (ABR), brain magnetic resonance imaging (MRI), electroretinogram (ERG), somatic evoked potentials (SEP), cerebrospinal fluid protein analysis, and parent questionnaires.

## Case Descriptions

### Patient 1

The first child is of Asian descent and was a product of a single, full term pregnancy and was 3.4 kg at birth. They were born *via* C-section due to failure of labor progression. They are the oldest of three siblings who were also diagnosed with Krabbe disease through NBS but have not yet developed signs of disease. The child reached all developmental milestones at an appropriate age and did not have any health issues throughout early childhood. However, following a school administered vision screening at 5 years and 11 months of age, it was determined that the child had reduced vision. At this time, they were referred to an ophthalmologist and prescribed glasses for vision correction. However, the glasses failed to make any difference in vision and the prescription was changed three times without any improvement. The patient was referred to a second ophthalmologist at 6 ½ years, where they presented with nystagmus, oscillopsia, and vision acuity of 20/500 on the right and 20/400 on the left. MRI of the brain a week later showed confluent T2 FLAIR hyperintensities in the periventricular region with posterior distribution and involvement of the optic radiation. The patient underwent further workup and was found to have two heterozygous pathogenic variants, p.L634S and p.R396W, in the GALC gene, confirming Krabbe diagnosis. Enzyme activity was found to be 0.08 nmol/h/mg of protein, and psychosine level was found to be 1.5 nmol/L. The child was transplanted with umbilical cord blood at 6 years and 9 months of age. As of the most recent follow up at 12 months post-transplantation, the patient's vision loss and changes in VEP have stabilized and there are no other signs of disease.

### Patient 2

The second patient is of Asian descent and was the 2.3 kg product of a 33 week long pregnancy complicated by placenta previa. The child had no neonatal complications and reached all developmental milestones in the appropriate age range. At 12 years of age, the patient noticed problems in vision, as they were having a difficult time seeing the blackboard at school. Initially, local physicians believed the vision difficulties may have been a result of an infectious process and they began a short course of steroids for 1 month. Partial recovery of vision was achieved to the extent that the child was able to identify objects at a distance of 1 m. However, when steroids failed to completely restore vision, a brain MRI and VEP were done, which revealed symmetrical white matter hyperintensities in both the parietal- occipital regions as well as splenium of the corpus callosum and bilateral prolonged P100 latencies respectively, suggestive of a leukodystrophy. Multiple follow-up studies, including ABR, NCV, and lumbar puncture were all normal, with no other indications of disease. Nevertheless, the patient underwent work-up for Krabbe disease, which revealed an enzyme activity of 0.12 nmol/h/mg of protein and pathogenic variants, p.Y319C and c.296+1G>T. Ophthalmological evaluation at this time revealed acuity of 2/180 in the left eye and 2/140 in the right, optic atrophy, and visual cortex/radiations disorder. Outside of vision impairment, a comprehensive neurodevelopmental assessment at this time revealed no other signs of disease other than slight difficulty with rapid alternating hand movements.

### Patient 3

The third patient is of Asian descent and was the 3.1 kg product of a full-term uncomplicated pregnancy. The child had no neonatal complications and reached all developmental milestones within an appropriate time frame. At 2 ½ years of age, the child's mom noticed that the patient could no longer identify objects that were four to five feet away and began relying on other senses to a greater extent. At this time, it was recommended that the patient have an ERG and VEP performed. The ophthalmologist interpreted the ERG as normal and the VEP as mildly abnormal. A follow-up brain MRI at 2 years and 7 months of age showed moderate confluent signal abnormalities involving the cerebral white matter, especially posteriorly affecting the occipital and parietal lobes. A spinal tap was also performed which revealed increased protein. At this time, the child was given a diagnosis of optic neuritis and treated with a course of intravenous steroids and a course of oral steroids. Following steroid treatment, there was a slight improvement in pupillary response but not vision. Because of the lack of improvement, the patient was revaluated by a geneticist at 3 years of age. Work up for Krabbe disease at this time revealed a GALC level of 0.04 nmol/h/mg and homozygosity for the p.Y319C variant. Notably, one of the patient's GALC alleles also included the well-known benign variant c.1685T>C_p.(I562T). Other than visual impairment, a comprehensive neurodevelopmental examination conducted following diagnosis revealed mild neuromuscular abnormalities including slight appendicular hypertonia and reduced deep tendon reflexes.

### Patient 4

The fourth patient is of Asian descent and was the product of a full-term uncomplicated pregnancy. The neonatal period was without difficulty, and the child met all developmental milestones in the appropriate time frame. The patient was proceeding without any health issues until 5 ½ years of age, when the child began to notice worsening vision and developed strabismus, for which corrective surgery was performed at 6 years of age. A VEP was administered at 6 years and 7 months of age, revealing reduced amplitudes and delayed latencies in the P100 wave, which were worse in the right eye. A mildly abnormal ERG was recorded at 7 years and 7 months of age, and a vision test at this time revealed bilateral visual acuity of 20/100. A subsequent MRI at 8 years of age showed posterior periventricular and corpus callosum white matter changes. At 8 years and 11 months, the patient underwent another vision test, which revealed substantial deterioration, with an acuity of 6/90 in the left eye and 9/120 in the right, combining to produce a bilateral acuity of 20/800. The persistent visual degeneration led to a second MRI at 9 years of age, which was characterized by marked progression of white matter changes and expansion of involvement into the internal capsule. The abnormal MRI prompted immediate diagnostic work up for a leukodystrophy. Results of testing came back with a GALC activity level of 0.10 nmol/h/mg and a homozygous p.Y319C genotype. Despite the patient's substantial vision impairment, the results of NCV and ABR testing at 9 years and 6 months were essentially normal, and the patient displayed no other signs of disease other than balance issues, thought to be related to poor vision.

### Patient 5

The fifth patient is of Asian descent and is the first cousin of patient 4 and was a 3.2 kg product of a full-term uncomplicated pregnancy. The child had no neonatal complications and reached all developmental milestones by an appropriate age. However, the patient had sporadic constipation between 1 and 2 years of life and was slow to gain weight after 2 years. Despite periodic constipation and slow weight gain, the patient remained healthy throughout early childhood. When the child was 3 years and 7 months old, their cousin (patient 4) was diagnosed with Krabbe disease, which prompted immediate diagnostic work up. Results of the work up confirmed a diagnosis of Krabbe disease with an enzyme activity level of 0.04 nmol/h/mg and a homozygous p.Y319C genotype. A comprehensive evaluation at this time revealed normal NCV, ABR, MRI, and SEP. However, VEPs were abnormal with reduced amplitude and delayed cortical latencies, suggestive of nerve dysfunction in the right eye. A second VEP at 4 years and 1 month showed worsening optic degeneration. Although the patient remains otherwise asymptomatic, their vision continues to worsen and is now severely impaired.

## Review of Literature

Because of the extreme rarity of Krabbe disease, efforts to uncover predictable genotype-phenotype correlations have been difficult. Thus, to date only a small number of potential genotype-phenotype correlations have been described and relatively well-established. These include the potential correlation of infantile-onset disease with the 30 kb del (11-17 exon deletion) variant when in a homozygous state or as a compound heterozygous state with non-sense or frameshift variants. Late-onset disease has also been observed in association with missense variants whether in a homozygous state [p.Gly286Asp], compound heterozygous state with two missense variations [p.Gly286Asp+p.Pro318Arg], or even when the same deletion [30 kb del (11-17 exon deletion)] was combined with the missense [p.Gly286Asp] variant (Table 1) (10, 11, 13–15). Nevertheless, it is imperative to note that, with the exception of homozygosity for the 30 kb del (11-17 exon deletion) variant—which is exclusively associated with the severe infantile phenotype—these potential correlations are based only on a small number of cases and may be subject to variability in clinical presentation.

**Table 1:** Potential genotype-phenotype correlations in Krabbe disease.

**Allele 1**	**Allele 2**	**Phenotype**	**References**
30 kb (11-17 exon deletion)	30 kb (11-17 exon deletion)	Infantile	(10)
30 kb (11-17 exon deletion)	Non-sense/frameshift	Infantile	(11)
30 kb (11-17 exon deletion)	p.Gly286Asp	Later onset	(6, 12)
p.Gly286Asp	p.Pro318Arg	Later onset	(11)
p.Leu634Ser	p.Leu634Ser	Later onset	(13, 14)

*This table describes previously identified genotype-phenotype correlations for Krabbe disease. This is based on expert consensus by Orsini et al. (10), published in Gene reviews*.

When specifically considering the variants present in the novel patients described in this report, we found six well-described previously reported cases that contained the p.Y319C allele. Of these six cases, all were compound heterozygotes. However, the age of onset varied greatly, with a range of 29 months to 23 years. Phenotypes for these patients included two adult onset, three juvenile onset, and one late-infantile onset. Of the five patients with sufficient clinical information, two (40%) presented initially with visual impairment (Table 2) (2, 3, 16). In addition, to these confirmed diagnoses of Krabbe disease, by conducting a large study of the New York State Newborn Screening Program, Orsini et al. (28) identified an additional 20 patients who were either compound heterozygotes or homozygotes for the p.Y319C allele. Based on the second variant and corresponding enzyme activity of these 20, 4 were categorized as high risk for developing disease, 14 as moderate risk, 1 as low risk, and 1 as no risk. However, none of these individuals had developed signs or symptoms of disease at the time of the study's publication and are therefore not included in Table 2 (28).

**Table 2:** Previously reported cases with pathogenic variants described in this case series.

**Publications**	**Zygosity**	**Allele 1**	**Allele 2**	**Age of onset**	**Origin**	**Phenotype**	**Initial Signs/symptoms**	**Vison impairment?**
Debs et al. (2)	Hetero	c.956A>G_p.Y319C	30 kb del (Exon 11-17)	~5 years	French	Juvenile	Bilateral vision loss	Yes
Debs et al. (2)	Hetero	c.956A>G_p.Y319C	30 kb del (Exon 11-17)	~12 years	French	Juvenile	Spastic paresis	No
Debs et al. (2)	Hetero	c.956A>G_p.Y319C	30 kb del (Exon 11-17)	~5 years	French	Juvenile	Impaired vision	Yes
Farina et al. (3)	Hetero	c.956A>G_p.Y319C	30 kb del (Exon 11-17)	~23 years	N/A	Adult	Walking difficulty	No
Farina et al. (3)	Hetero	c.956A>G_p.Y319C	30 kb del (Exon 11-17)	~23 years	N/A	Adult	Walking difficulty	No
Duffner et al. (16)	Hetero	c.956A>G p.Y319C	c.860 1G>A	29 months	N/A	Late-infantile	N/A	N/A
Szymańska et al. (17)	Hetero	c.1186C>T_p.R396W	30 kb del (Exon 11-17)	13 months	Polish	Late-infantile	Swallowing difficulty	No
Duffner et al. (18)	Hetero	c.1186C>T p.R396W	30 kb del (Exon 11-17)	N/A	N/A	Infantile	N/A	N/A
Tappino et al. (11)	Hetero	c.1186C>T p.R396W	c.749T>C p.I250T	11 months	N/A	Infantile	Hypotonia	N/A
Naidu et al. (19)	Homo	c.1186C>T p.R396W	c.1186C>T p.R396W	11 months	N/A	Infantile	Motor regression	No
Madsen et al. (20)	Homo	c.1186C>T p.R396W	c.1186C>T p.R396W	11 months	Non-European	Infantile	Motor regression	No
Zhao et al. (21)	Hetero	c.1901T>C_p.L634S	c.953C>T_p.P318L	N/A	Chinese	N/A	Limb movement disorder	No
Zhao et al. (21)	Hetero	c.1901T>C_p.L634S	Unknown	~20 years	Chinese	Adult	Aphasia	No
Zhao et al. (21)	Hetero	c.1901T>C_p.L634S	c.195+1 G>A	8 years 10 months	Chinese	Juvenile	Vision impairment	Yes
Zhao et al. (21)	Hetero	c.1901T>C_p.L634S	c.379C>T_p.R129X	2 years	Chinese	Late-infantile	Motor regression	No
Zhao et al. (21)	Hetero	c.1901T>C_p.L634S	c.1048 T>G_p.F350V	10 months	Chinese	Infantile	Developmental delay	Yes
Tappino et al. (11)	Hetero	c.1901T>C_p.L634S	c.1489+1G>A	7 months	N/A	Infantile	Spasticity	No
Zhang et al. (14)	Hetero	c.1901T>C_p.L634S	c.1901delT	20 years	Chinese	Adult	Limb weakness	No
Satoh et al. (22)	Homo	c.1901T>C_p.L634S	c.1901T>C_p.L634S	38 years	N/A	Adult	Spastic paresis	No
Xu et al. (23)	Hetero	c.1901T>C_p.L634S	p.P318A	8 months	Japanese	Infantile	N/A	N/A
Furuya et al. (6)	Hetero	c.1901T>C_p.L634S	c.582+5G>A	~20 years	Japanese	Adult	Spastic paresis	No
Meng et al. (24)	Hetero	c.1901T>C_p.L634S	c.1005C>G_p.Y335X	~40 years	Chinese	Adult	Spastic paresis	Yes
Hossain et al. (13)	Hetero	c.1901T>C_p.L634S	Unknown	~56 years	Japanese	Adult	N/A	N/A
Hossain et al. (13)	Hetero	c.1901T>C_p.L634S	c.1985G>C_p.G662A	~35 years	Japanese	Adult	N/A	N/A
Hossain et al. (13)	Hetero	c.1901T>C_p.L634S	Unknown	~14 years	Japanese	Adult	N/A	N/A
Hossain et al. (13)	Hetero	c.1901T>C_p.L634S	c.904C>G_p.P302A	~3 years	Japanese	Late-infantile	N/A	N/A
Hossain et al. (13)	Hetero	c.1901T>C_p.L634S	Unknown	~2 years	Japanese	Late-infantile	N/A	N/A
Hossain et al. (13)	Hetero	c.1901T>C_p.L634S	c.1719dupT	14 months	Japanese	Late-infantile	N/A	N/A
Hossain et al. (13)	Hetero	c.1901T>C_p.L634S	c.1719dupT	11 months	Japanese	Infantile	N/A	N/A
Yoshimura et al. (25)	Hetero	c.1901T>C_p.L634S	c.683_694delinsCTC	11 months	Japanese	Infantile	Spastic paresis	Yes
Lim et al. (26)	Hetero	c.1901T>C_p.L634S	c.1687A>T:p.K563X	~12 years	N/A	Juvenile	Walking difficulty	Yes
Xia et al. (27)	Homo	c.1901T>C_p.L634S	c.1901T>C_p.L634S	~22 years	Chinese	Adult	Seizures	No
Xie et al. (27)	Hetero	c.1901T>C_p.L634S	c.1321C>T p.Q441X	~12 years	Chinese	Juvenile	Ataxia and spastic paresis	N/A
Xie et al. (27)	Herero	c.1901T>C_p.L634S	c.2041G>A p.V681M	~26 years	Chinese	Adult	Spastic paresis	N/A

*This table includes all previously published reports of cases involving c.296+1G>T, c.956A>G_p.Y319C, and c.1186C>T_p.R396W. Columns with N/A means that the original publication did not include the pertinent information*.

In comparison, cases presenting with the p.L634S variant were much more common throughout the literature. Of the 23 previously published cases, 17 were compound heterozygotes, 2 were homozygous, and 4 failed to present information on the second variant. In general, p.L634S was associated with later onset disease, of which 10 cases presented with adult onset, three presented with juvenile onset, four presented with late-infantile onset, and four presented with infantile onset. Nevertheless, the range of initial symptoms varied greatly, with the earliest onset occurring at 7 months and the latest occurring at 56 years. Notably, the two homozygous patients did not develop signs or symptoms until 22 and 38 years of age. Of the 13 patients with sufficient clinical information, five (38%) presented with significant visual impairment (Table 2) (6, 11, 13, 14, 21–27, 29).

As for the p.R396W variant, reports are relatively scarce in the literature, totaling five. One of the previously described individuals presented at 13 months of age with swallowing difficulties and temperature dysregulation. The patient died shortly after diagnosis, at 15 months, with no mention of vision loss (17). Three other cases with the p.R396W variant all presented at 11 months of age with neuromuscular issues or motor regression, before passing at the ages of 18 months, 35 months, and one where age of death was not reported (11, 19, 20). The last case of the p.R396W reported in the literature contained minimal clinical data, however, the patient was reported to have infantile onset and only survived to the age of 13 months (18). The final variant found in this series, c.296+1G>T, has not been reported in any prior publications and, thus, was described here for the first time.

## Discussion

This case series described five late onset patients with Krabbe disease (four juvenile and one late-infantile) and provided a brief literature review of pertinent cases. Of clinical interest, all five patients were of Asian descent—a population where late-onset phenotypes may be more common in comparison to populations of European and African descent—and presented initially with vision impairment (21). It is important to note that four of the patients were originally ascribed a misdiagnosis or experienced a significant delay between initial symptoms and the final diagnosis. In contrast to most typical cases of Krabbe disease, patients did not present with marked appendicular spasticity (only patient 3 presented with mildly increased tone) or axial hypotonia, and all five reached developmental milestones within the normal time frame. For neurophysiological testing, no patient showed abnormalities in NCV, ABR, or SEP. However, abnormalities in VEP or ERG were seen in all patients and were one of the first markers of disease, along with abnormal MRI findings. The one patient who underwent transplantation following diagnosis demonstrated stabilization in vision and showed no further changes on VEP.

Vision loss is often assumed to be a common clinical feature of Krabbe disease that develops in the late stages of disease, well after patients have lost most functional skills. However, this case series demonstrates that for certain variants, severe vision impairment manifests prior to the onset of all other symptoms. Four of the patients in the study tested positive for the p.Y319C variant, three of which were homozygous (patient 3, 4, 5) and one which was a compound heterozygote with the variant, c.296+1G>T, on the second allele (patient 2) (Table 3). Recent functional studies have shown that the p.Y319C substitution leads to protein misfolding, resulting in lack of enzyme secretion, inability to be proteolytically processed, and retention in the endoplasmic reticulum, preventing localization to the lysosome (30). Since first identified by Farina et al. (3), the p.Y319C variant has been described as a predominately later onset variant, even when combined with the classical 30 kb del (exon 11-17) infantile variant (2, 3, 16). In addition, according to the gnomAD v 2.1.1, the p.Y319C variant is particularly common in the Southeast Asian population, with a reported prevalence of ~1% in tested individuals, compared to 0.1% in the general population. Despite characterization of p.Y319C as predictive of later onset, previous studies have reported a large degree of variation in the clinical profile of children and young adults who test positive for the variant, even within the same family (2). However, the reported variability may be a result of secondary deficits that manifest as a result of vision impairment, since, when combining previous cases from the literature with the four new cases presented here, we find a 67% (6/9) prevalence for vision loss. For instance, vision impairment can cause balance issues, as in patient 4, or psychometric regression, which can be misinterpreted as upper motor tract or frontal lobe involvement without a comprehensive ophthalmological evaluation or VEP. Thus, the four patients with p.Y319C in this study and identification of severe or complete vision loss help to unify and expand our understanding of the genotype-phenotype correlation for p.Y319C. Furthermore, the fact that this study provides the first detailed accounts of the disease course in three separate patients homozygous for the p.Y319C variant, allows us to provide unique insight into the influence that the variant has on clinical presentation. It is also important to note that while the benign p.(I562T) variant is often reported in GALC gene sequencing, the base-pair substitution is known to be non-pathogenic on its own. Therefore, it is interesting to note that the one patient homozygous for p.Y319C, who also possessed one copy of the p.(I562T), presented earlier than the other two homozygotes. Thus, p.(I562T) may function as a genetic modifier of the p.Y319C variant (31). As for the c.296+1G>T variant found in combination with p.Y319C in patient 2, the variant has never been reported before in the literature but is likely to affect the splicing of intron three and is, thus, likely pathogenic in nature. Moreover, because patient 2 did not have onset until 12 years of age, c.296+1G>T may correspond to a late onset phenotype.

**Table 3:** Genotype and phenotype for patients first described in this report.

**Patient**	**Phenotype**	**Allele 1**	**Allele 2**
1	Juvenile	c.1186C>T_p.R396W	c.1901T>C_p.L634S
2	Juvenile	c.956A>G_p.Y319C	c.296+1G>T
3	Late-infantile	c.956A>G_p.Y319C	c.956A>G_p.Y319C
4	Juvenile	c.956A>G_p.Y319C	c.956A>G_p.Y319C
5	Juvenile	c.956A>G_p.Y319C	c.956A>G_p.Y319C

*This table displays the genotype and phenotype for the 5 new patients described in this case series. In addition to the patients, parents were tested to confirm inheritance from parent to patient. Furthermore, all variants were confirmed to be in trans because each parent carried one of the alleles present in their children*.

Although patient 1 presented similarly to the other patients in the case report, they did not have the p.Y319C variant on either allele. Reports of the p.R396W variant are relatively scarce in the literature. Of the five previously reported patients with the variant, all five had onset within the first 13 months of life and none survived past 35 months (11, 17–20). In contrast, the p.L634S variant is significantly more common and is consistently associated with later onset phenotypes. Commonly found in Asian populations, the variant is frequently associated with vision loss. For example, two of the five patients reported to have the variant in a recent cases series by Zhao et al. (21) were reported to present with vision loss early in disease progression, although there was no information on VEP (21). When we combine the one new case with p.L634S with previous cases reported in Table 2, we find a prevalence of 43% (6/14).

Taken together, vision loss is often described as a common feature of Krabbe disease. Nevertheless, the prevalence of vision loss is likely overestimated, and the timing of its onset will vary depending on the patient's genotype. For example, with infantile onset, vision loss is likely to be a manifestation of advanced disease, with other signs and symptoms emerging before vision loss (7, 8). As such, infantile patients are too advanced to undergo transplantation once they are blind. In contrast, patients with a later onset phenotype are more likely to experience vision loss as an early manifestation of disease, depending on their genotype, and may still be eligible for transplantation, as observed with patient 1. Thus, it is extremely important that patients with p.L634S and p.Y319C be closely monitored for abnormal vision and VEP results, as such abnormalities should prompt immediate evaluation and work-up for HSCT.

## Conclusions

Major limitations of this study include the small sample size, retrospective design, lack of a direct control group, need for replication from another independent cohort, and the fact that two patients who were homozygous for p.Y319C were relatives. Nevertheless, this knowledge will become increasingly important as physicians become cognizant of what signs and symptoms to look for when monitoring children identified through NBS who have the pathogenic variants described in this report.

## Data Availability Statement

The raw data supporting the conclusions of this article will be made available by the authors, without undue reservation.

## Ethics Statement

All research was conducted and parental consent obtained with the approval of the institutional review boards of the University of Pittsburgh (IRB-PRO11050036) and University of North Carolina (IRB-08-0237). Written informed consent to participate in this study was provided by the participants' legal guardian/next of kin. Written informed consent was obtained from the minor(s)' legal guardian/next of kin for the publication of any potentially identifiable images or data included in this article.

## Author Contributions

NB collected the data and drafted the majority of the manuscript. MB-Q drafted a portion of the manuscript. ME was the treating physician, collected the data as part of an IRB approved longitudinal study, and was responsible for the overall study. All authors contributed to the article and approved the submitted version.

## Conflict of Interest

The authors declare that the research was conducted in the absence of any commercial or financial relationships that could be construed as a potential conflict of interest.

## References

[1] Wenger D Rafi M Luzi P. Molecular genetics of Krabbe disease (globoid cell leukodystrophy): diagnostic and clinical implications. Hum Mutat. (1997) 10:268–79. 9338580 10.1002/(SICI)1098-1004(1997)10:4<268::AID-HUMU2>3.0.CO;2-D

[2] Debs R Froissart R Aubourg P Papeix C Douillard C Degos B. Krabbe disease in adults: phenotypic and genotypic update from a series of 11 cases and a review. J Inherit Metab Dis. (2013) 36:859–68. 10.1007/s10545-012-9560-4 23197103

[3] Farina L Bizzi A Finocchiaro G Pareyson D Sghirlanzoni A Bertagnolio B. MR imaging and proton MR spectroscopy in adult Krabbe disease. AJNR. (2000) 21:1478–82. 11003282 PMC7974033

[4] Knaap M Valk J Globoid cell leukodystrophy: Krabbe disease. In: Heilmann U editors Magnetic Resonance of Myelination and Myelin Disorders. Heidelberg: Springer (2005). p. 87–95.

[5] Duffner P Caggana M Orsini J Wenger D Patterson M Crosley C. Newborn screening for Krabbe disease: the New York state model. Pediatr Neurol. (2009) 40:245–52; discussion 253–5. 10.1016/j.pediatrneurol.2008.11.010 19302934

[6] Furuya H Kukuta Y Nagano S Sakai Y Yamashita Y Fukuyama H. Adult onset globoid cell leukodystrophy (Krabbe disease): analysis of galactosylceramidase cDNA from four Japanese patients. Hum Genet. (1997) 100:450–6. 10.1007/s004390050532 9272171

[7] Bascou NA DeRenzo A Poe MD Escolar ML. A prospective natural history study of Krabbe disease in a patient cohort with onset between 6 months and 3 years of life. Orphanet J Rare Dis. (2018) 13:126. 10.1186/s13023-018-0872-9 30089515 PMC6083585

[8] Beltran-Quintero ML Bascou NA Poe MD Wenger DA Saavedra-Matiz CA Nichols MJ. Early progression of Krabbe disease in patients with symptom onset between 0 and 5 months. Orphanet J Rare Dis. (2019) 14:46. 10.1186/s13023-019-1018-4 30777126 PMC6378723

[9] Beltran Quintero ML Roosen-Marcos MC Cladis FP Poe MD Escolar ML. General anesthesia safety in progressive leukodystrophies: A retrospective study of patients with Krabbe disease and metachromatic leukodystrophy. Paediatr Anaesth. (2019) 29:1053–059. 10.1111/pan.13714 31359511

[10] Orsini JJ Escolar ML Wasserstein MP Caggana M Krabbe disease. In: Adam MP editor. GeneReviews^®^[Internet]. Seattle, WA: University of Washington (2018). p. 1–21. 20301416

[11] Tappino B Biancheri R Mort M Regis S Corsolini F Rossi A. Identification and characterization of 15 novel GALC gene mutations causing Krabbe disease. Hum Mutat. (2010) 31:E1894–915. 10.1002/humu.21367 20886637 PMC3052420

[12] De Gasperi R Gama Sosa MA Sartorato E Battistini S Raghavan S Kolodny EH. Molecular basis of late-life globoid cell leukodystrophy. Hum Mutat. (1999) 14:256–62. 10.1002/(SICI)1098-1004(1999)14:3<256::AID-HUMU9>3.0.CO;2-6 10477434

[13] Hossain MA Otomo T Saito S Ohno K Sakuraba H Hamada Y. Late-onset Krabbe disease is predominant in Japan and its mutant precursor protein undergoes more effective processing than the infantile-onset form. Gene. (2014) 534:144–54. 10.1016/j.gene.2013.11.003 24252386

[14] Zhang T Yan C Ji K Lin P Chi L Zhao X. Adult-onset Krabbe disease in two generations of a Chinese family. Ann Transl Med. (2018) 6:174. 10.21037/atm.2018.04.30 29951496 PMC5994525

[15] Luzi P Rafi MA Wenger DA Multiple mutations in the GALC gene in a patient with adult-onset Krabbe disease. Ann Neurol. (1996) 40:116–9. 10.1002/ana.410400119 8687180

[16] Duffner PK Barczykowski A Kay DM Jalal K Yan L Abdelhalim A. Later onset phenotypes of Krabbe disease: results of the world-wide registry. Pediatr Neurol. (2012) 46:298–306. 10.1016/j.pediatrneurol.2012.02.023 22520351

[17] Szymańska K Ługowska A Laure-Kamionowska M Bekiesińska-Figatowska M Gieruszczak-Białek D Musielak M Diagnostic difficulties in Krabbe disease: a report of two cases and review of literature. Folia Neuropathol. (2012) 50:346–56. 10.5114/fn.2012.32364 23319190

[18] Duffner PK Barczykowski A Jalal K Yan L Kay DM Carter RL. Early infantile Krabbe disease: results of the world-wide Krabbe registry. Pediatr Neurol. (2011) 45:141–8. 10.1016/j.pediatrneurol.2011.05.007 21824559

[19] Naidu S Hofmann KJ Moser HW Maumenee IH Wenger DA. Galactosylceramide-β-galactosidase deficiency in association with cherry red spot. Neuropediatrics. (1988) 19:46–8. 10.1055/s-2008-1052400 3362311

[20] Madsen AM Wibrand F Lund AM Ek J Dunø M Østergaard E. Genotype and phenotype classification of 29 patients affected by Krabbe disease. JIMD Rep. (2019) 46:35–45. 10.1002/jmd2.12007 31240153 PMC6498822

[21] Zhao S Zhan X Wang Y Ye J Han L Qiu W. Large-scale study of clinical and biochemical characteristics of Chinese patients diagnosed with Krabbe disease. Clin Genet. (2018) 93:248–54. 10.1111/cge.13071 28598007

[22] Satoh JI Tokumoto H Kurohara K Yukitake M Matsui M Kuroda Y Adult-onset Krabbe disease with homozygous T1853C mutation in the galactocerebrosidase gene. Neurology. (1997) 49:1392–9. 10.1212/WNL.49.5.1392 9371928

[23] Xu C Sakai N Taniike M Inui K Ozono K. Six novel mutations detected in the GALC gene in 17 Japanese patients with Krabbe disease, and new genotype–phenotype correlation. J Hum Genet. (2006) 51:548–54. 10.1007/s10038-006-0396-3 16607461

[24] Meng X Li Y Lian Y Li Y Du L Xie N Wang C. A new compound heterozygous mutation in adult-onset Krabbe disease. Int J Neurosci. (2020) 120:1-5. 10.1080/00207454.2020.1731504 32064984

[25] Yoshimura A Kibe T Irahara K Sakai N Yokochi K. Predominant corticospinal tract involvement in a late infant with Krabbe disease. Jap Clin Med. (2016) 7:JCM-S40470. 10.4137/JCM.S40470 27679535 PMC5027888

[26] Lim SM Choi BO Oh SI Choi WJ Oh KW Nahm M. Patient fibroblasts-derived induced neurons demonstrate autonomous neuronal defects in adult-onset Krabbe disease. Oncotarget. (2016) 7:74496. 10.18632/oncotarget.12812 27780934 PMC5342682

[27] Xia Z Wenwen Y Xianfeng Y Panpan H Xiaoqun Z Zhongwu S. Adult-onset Krabbe disease due to a homozygous GALC mutation without abnormal signals on an MRI in a consanguineous family: a case report. Mol Genet Genomic Med. (2020) 8:e1407. 10.1002/mgg3.1407 32677356 PMC7507702

[28] Orsini JJ Kay DM Saavedra-Matiz CA Wenger DA Duffner PK Erbe RW. Newborn screening for Krabbe disease in New York state: the first eight years' experience. Genet Med. (2016) 18:239–48. 10.1038/gim.2015.211 26795590

[29] Xie JJ Ni W Wei Q Ma H Bai G Shen Y. New clinical characteristics and novel pathogenic variants of patients with hereditary leukodystrophies. CNS Neurosci Ther. (2020) 26:567–75. 10.1111/cns.13284 31885218 PMC7163788

[30] Spratley SJ Hill CH Viuff AH Edgar JR Skjødt K Deane JE. Molecular mechanisms of disease pathogenesis differ in Krabbe Disease variants. Traffic. (2016) 17:908–22. 10.1111/tra.12404 27126738 PMC4949656

[31] Wenger DA Luzi P Rafi MA Krabbe disease: are certain mutations disease-causing only when specific polymorphisms are present or when inherited in trans with specific second mutations? Mol Genet Metab. (2013) 111:307–8. 10.1016/j.ymgme.2013.12.009 24388568

